# Increased Zinc and Manganese in Parallel with Neurodegeneration, Synaptic Protein Changes and Activation of Akt/GSK3 Signaling in Ovine CLN6 Neuronal Ceroid Lipofuscinosis

**DOI:** 10.1371/journal.pone.0058644

**Published:** 2013-03-14

**Authors:** Katja M. Kanninen, Alexandra Grubman, Jodi Meyerowitz, Clare Duncan, Jiang-Li Tan, Sarah J. Parker, Peter J. Crouch, Brett M. Paterson, James L. Hickey, Paul S. Donnelly, Irene Volitakis, Imke Tammen, David N. Palmer, Anthony R. White

**Affiliations:** 1 Department of Pathology, The University of Melbourne, Victoria, Australia; 2 School of Chemistry and Bio21 Molecular Science and Biotechnology Institute, The University of Melbourne, Victoria, Australia; 3 Florey Institute of Neuroscience and Mental Health, The University of Melbourne, Victoria, Australia; 4 ReproGen, Faculty of Veterinary Science, The University of Sydney, Camden, New South Wales, Australia; 5 Faculty of Agriculture and Life Sciences, Lincoln University, Lincoln, Canterbury, New Zealand; Hertie Institute for Clinical Brain Research and German Center for Neurodegenerative Diseases, Germany

## Abstract

Mutations in the *CLN6* gene cause a variant late infantile form of neuronal ceroid lipofuscinosis (NCL; Batten disease). CLN6 loss leads to disease clinically characterized by vision impairment, motor and cognitive dysfunction, and seizures. Accumulating evidence suggests that alterations in metal homeostasis and cellular signaling pathways are implicated in several neurodegenerative and developmental disorders, yet little is known about their role in the NCLs. To explore the disease mechanisms of CLN6 NCL, metal concentrations and expression of proteins implicated in cellular signaling pathways were assessed in brain tissue from South Hampshire and Merino CLN6 sheep. Analyses revealed increased zinc and manganese concentrations in affected sheep brain in those regions where neuroinflammation and neurodegeneration first occur. Synaptic proteins, the metal-binding protein metallothionein, and the Akt/GSK3 and ERK/MAPK cellular signaling pathways were also altered. These results demonstrate that altered metal concentrations, synaptic protein changes, and aberrant modulation of cellular signaling pathways are characteristic features in the CLN6 ovine form of NCL.

## Introduction

Neuronal ceroid lipofuscinoses (NCLs; Batten disease) are a group of inherited, progressive neurodegenerative diseases of childhood (recently reviewed in [1]. They represent the most common neurodegenerative disorder of children with an incidence of up to 1∶12500 [2]. The estimates of incidence are rapidly increasing with better diagnosis. There are at least eight different childhood forms of NCL, distinguished by the age of onset and the genetic defect, and three adult forms of the disease [3]–[5]. Although traditionally described by the ages of onset, all NCL forms share similar clinical and pathological features. Clinical symptoms include vision impairment, motor dysfunction, cognitive dysfunction and seizures, and ultimately lead to premature death. Pathological features of the disease include neuronal degeneration and glial activation. Fluorescent lysosome-derived storage bodies accumulate in most tissues. These contain protein, specifically subunit c of mitochondrial ATP synthase in CLN2, CLN3, CLN5, CLN6, CLN7 and CLN8, and SAPs A and D in the CLN1 and CLN10 forms [6]–[8]. Four of the causative NCL genes, CLN1, CLN2, CLN5 and CLN10, code for soluble lysosomal proteins and the rest are membrane bound, associated with the endosomal/lysosomal organelles, endoplasmic reticulum (ER), and synaptic vesicles [9]. The physiological functions of all the NCL proteins and the pathophysiological mechanisms underlying the NCLs remain unclear.

The CLN6 protein is a highly conserved transmembrane protein of unknown function that has been localized to the ER [10], [11]. As studies in human NCLs are restricted, naturally occurring animal models of the disease are an important resource for deciphering the disease mechanisms. Sheep are a well-characterized, naturally occurring animal model for CLN6 disease in which the phenotype resembles the human disease [12]–[14]. They have a complex central nervous system (CNS) and a prolonged disease progression similar to human CLN6 disease, easily observable at the age of 10–14 months, increasing blindness being the leading clinical sign. Neuropathology is characterized by cortical neurodegeneration, which begins in the visual, parietal and occipital cortex at four months of age, then spreads to other regions [15]. This is preceded by activation of astrocytes and microglia, which begins perinatally in the same regions before spreading [16]. The balance of interneuron populations changes [17] while some regions of the brain remain unaffected [18]. On the other hand, storage body accumulation occurs similarly across all brain regions, and is notable in cells not subject to neurodegeneration, indicating that it does not play a central role in the pathogenic cascade [15].

Accumulating evidence suggests that altered metal homeostasis is a common pathological feature of several neurodegenerative diseases, including Alzheimer's disease, Parkinson's disease, and motor neuron disease (reviewed in [19], [20] but also of developmental disorders such as autistic spectrum disorder [21] and neurodegeneration with brain iron accumulation [22]. Zinc, in particular, is an important transition metal linked with physiology and pathology in the CNS (reviewed in [23]). Zinc functions as an essential component of many proteins and acts as a signaling messenger at synapses. It has an important role in intracellular signaling pathways that regulate both cell survival and death. Cells are vulnerable to changes in zinc concentration, which are regulated by transporter proteins [23] and by metal-binding proteins such as metallothionein (MT) [24].

It is increasingly evident that metals play a complex role in neurodegenerative disease, yet there is little knowledge of the role of metals in NCL affected brains. Changes in some metal concentrations, including iron and zinc in blood cells from patients with NCL were reported [25]. In a larger study of NCL patients, no association between ‘loosely bound’ iron or copper levels in cerebrospinal fluid could be found [26]. Early studies on the metal contents of storage material from CLN6 sheep revealed no obvious association between metal contents and disease progress [27]. They varied between storage material from different tissues in line with the normal role of lysosomes as storage depots for metals, indicating the lysosomal origin of the storage material. Changes in the metal contents of retina and retinal pigment epithelial cells of CLN6 sheep have been reported, and an association with storage material accumulation and altered manganese concentrations with increasing photoreceptor cell loss suggested [28]. General brain metal contents in CLN6 and unaffected animals or human NCL patients have not been compared.

In the present study, we have begun to investigate the role of biometals in the South Hampshire and Merino ovine models of CLN6 disease. The most striking finding was a substantial zinc, and to a lesser extent manganese, accumulation in specific regions of the affected brain. This accumulation was evident at 12–14 months of age as were aberrant activation of cellular signaling cascades and changes to synaptic proteins. These data suggest that altered brain metal concentrations and disturbances of cellular signaling pathways are a feature of ovine CLN6 disease.

## Materials and Methods

### Animals and materials

Sheep were maintained under standard New Zealand or Australian pasture conditions on a University research farm. All animal procedures were carried out according to NIH guidelines and the New Zealand Animal Welfare Act (1999) or the New South Wales Animal Research Act (1985) [13]. All animal work was performed using standard operating procedures and this study was approved by the Lincoln University Animal Ethics Committee. CLN6 affected Merino sheep have a c184C>T mutation and the South Hampshire sheep display reduced CLN6 mRNA. Unaffected South Hampshire and Merino sheep, and CLN5 (Borderdale) heterozygous (CLN5+/−, unaffected) animals [29] were used as controls. Brain samples were collected from five 12–14 month-old control and seven CLN6 affected sheep. At post mortem, each brain was dissected into the occipital lobe, parietal lobe, frontal lobe, thalamus, cerebellum and brain stem, and immediately frozen. All reagents used were analytical grade.

### Metal analyses

The metal content in six regions of the sheep brain was measured using inductively coupled plasma mass-spectrometry (ICP-MS) as previously described [30]. Weighed tissue pieces were lyophilized, digested in 150–400 µl of 65% nitric acid (Merck, Kilsyth, Victoria, Australia) overnight, and heated for a further 20 min at 90°C. Then 400 µl of BDH prolabo 30% hydrogen peroxide (VWR, Murrarie, QLD, Australia) was added to each sample and incubated at room temperature for 30 min, then for a further 15 min at 70°C. All samples were diluted 1∶20 with 1% nitric acid before being measured in an Agilent 7700 series ICP-MS instrument (Agilent Technologies, Santa Clara, CA, USA) using a helium reaction gas cell. The instrument was calibrated using 0, 5, 10, 50, and 100 ppb of certified multi-element ICP-MS standard calibration solutions (Accustandard, New Haven, CT, USA) for a range of elements. 200 ppb of yttrium (Y89) was used as an internal control. Samples were analyzed in triplicate and median values were used for analyses. The results are expressed as micrograms of metal per gram of wet weight (µg/g).

### Western blots

Brain tissues were homogenized with a Dounce tissue grinder in 5 volumes of Novagen Phosphosafe extraction reagent (Merck) containing protease inhibitor cocktail (Roche). The protein concentrations of supernatants after centrifugation (12,000 *g*, 5 min, 4°C) were measured with a Pierce BCA assay kit (Thermo Scientific, Scoresby, Victoria, Australia) according to manufacturer's instructions. Cortical neurons were harvested into Phosphosafe extraction reagent containing protease inhibitor cocktail as above. Proteins were separated on 12% SDS-PAGE tris-glycine gels, electrophoretically transferred to PVDF membranes for immunoblotting, and blocked with 4% skim milk in phosphate buffered saline pH 7.4 (PBS)-0.05% Tween. Membranes were probed with polyclonal antisera, all from Cell Signaling Technology (Danvers, MA, USA), for total- or phospho- ERK1/2, Akt, GSK3, p38, MAPK, syntaxin-6, GAPDH, ß-tubulin and ß-actin. The synaptophysin antiserum was purchased from Millipore (Billerica, MA, USA) and the metallothionein antibody was from Abcam (Cambridge, MA, USA). The horseradish peroxidase-conjugated anti-rabbit or anti-mouse secondary antiserum (Cell Signaling Technology) was used at 1∶5,000 dilution. Membranes were developed by chemiluminescence using an Amersham ECL Advance Western blotting detection kit, (GE Healthcare, Rydalmere, NSW, Australia) and imaged on a Fujifilm LAS3000 Imager (Berthold, Bundoora, Australia). Western blots were subjected to densitometry analysis using ImageJ software. Target band intensity was compared to the intensity of control bands. Densitometric quantification of phosphorylated Akt and phosphorylated GSK3ß were performed as follows: both the phosphorylated form and total form were first normalized to the loading control, GAPDH. The ratio of normalized phospho-protein to total protein was then shown in the figures. Data are shown as means ± S.D.

### Statistical analyses

The co-relations of genotypes and brain regions to changes in metal concentrations were examined using 2-way ANOVA in SPSS 18.0 software (Softonic, Barcelona, Spain). If either statistically significant main effects or interactions of brain region and genotype were detected, simple main effects tests were performed to determine those brain regions in which the genotypes had different metal concentrations. Student's t-test in GraphPad Prism 5d (GraphPad Software, La Jolla, CA, USA) was used for densitometry of Western blots. Data are shown as means ± S.D.

## Results

### Elevated zinc and manganese in CLN6 ovine brain

**Figure 1:**
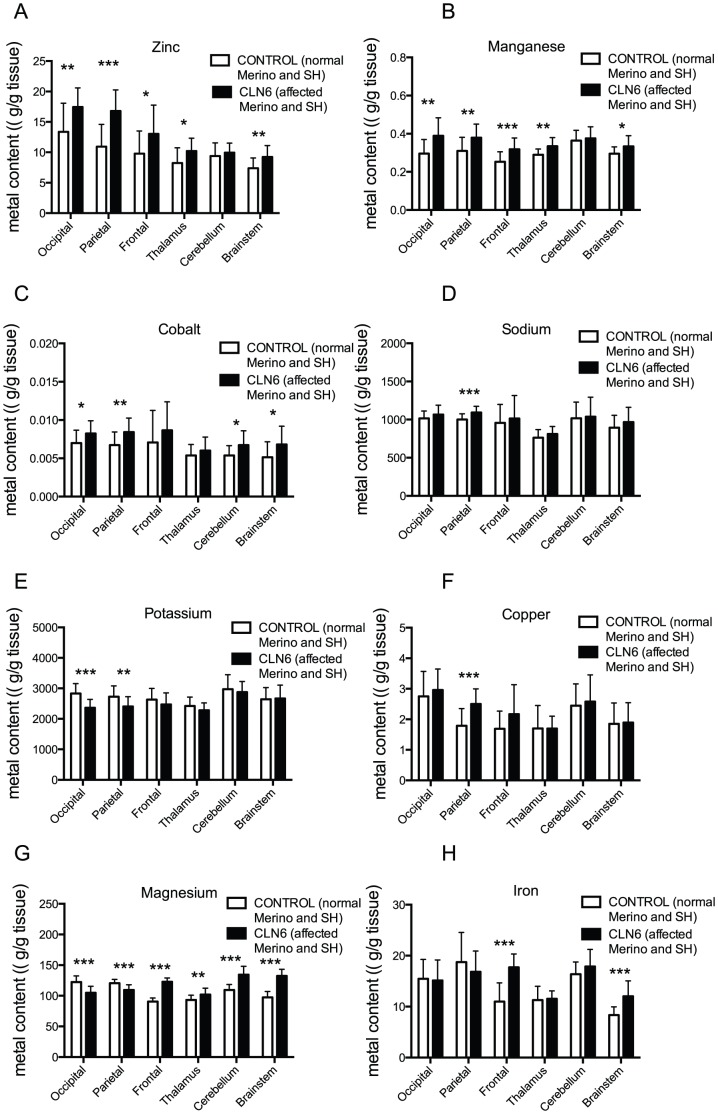
Altered metal concentrations in CLN6 disease affected brain. Metal concentrations in the occipital lobe, parietal lobe, frontal lobe, thalamus, cerebellum, and brainstem of 12–14 month old Merino and South Hampshire (SH) CLN6 and Merino and South Hampshire control sheep were measured using ICP-MS. The metal concentrations in each brain region are expressed relative to control metal concentrations. Error bars represent S.D. values. ***p<0.001, **p<0.01, *p<0.05.

The metal concentrations in the occipital lobe, parietal lobe, frontal lobe, thalamus, cerebellum, and brainstem of 12–14 month old CLN6 affected (Merino and South Hampshire background), control (Merino and South Hampshire background), and heterozygous CLN5 (Borderdale background) sheep were measured using ICP-MS. This is the age of onset for readily detectable symptomatic changes in the affected animals. Eight metal concentrations were significantly different in the CLN6 affected ovine brain samples when compared to controls (Fig. 1). Others were similar for controls and CLN6 sheep (Fig. S1) and the concentrations of a further 11 metals (B, Ni, Ge, Se Ga, Ge, Mo, Cd, Ba, W and Pb) were at or below the limit of detection.

Zinc concentrations were significantly higher in the CLN6 affected sheep when compared to controls in all brain regions analyzed except cerebellum (Fig. 1A). Accumulation was most pronounced in the occipital and parietal lobes of affected animals. Interestingly, the metal differences between brain regions within both control and affected animals were also significant (p<0.001).

The amount of manganese was also increased in the occipital, parietal, and frontal lobes, and thalamus and brainstem of the 12–14 month old affected compared to control sheep (Fig. 1B). Cobalt concentrations changed with both mutations (p<0.001) and brain region (p<0.001) (Fig. 1C). Sodium and potassium concentrations, two ions essential for maintenance of the membrane potential, also changed in the CLN6 sheep brain (Fig. 1D and E). Out of the 30 metals analyzed potassium (Fig. 1E) and magnesium (Fig. 1G) were the sole elements in which a reduction was observed in the brains of CLN6 affected sheep. Copper concentrations were increased in the parietal lobe of affected animals (Fig. 1F) and iron was increased in the frontal lobe and brain stem of CLN6 sheep (Fig. 1H). Magnesium concentrations were altered in all brain regions of CLN6 animals (Fig. 1G).

As a further control, metal concentrations were also measured in CLN5+/− brains (on a Borderdale background). CLN5 +/− sheep do not become disease-affected. The metal concentrations in CLN5 heterozygotes were not significantly different when compared to control South Hampshire or Merino brains (data not shown) providing further evidence that breed alone does not significantly affect brain metal concentrations at this age.

### Elevated metallothionein in CLN6 ovine brain

**Figure 2:**
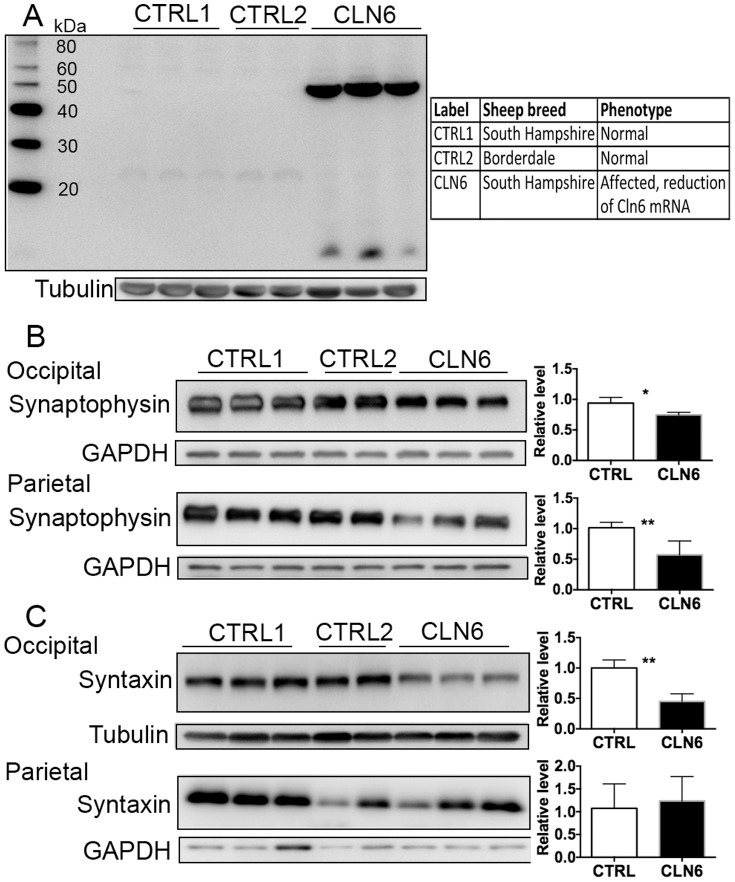
Reduction of metallothionein and synaptic proteins in CLN6 affected brain. (A) Occipital lobe homogenate from control and CLN6 affected sheep was immunoblotted with an antibody for metallothionein I/II and β–tubulin as loading control. (B) Five micrograms of homogenate from occipital and parietal lobe from 12–14 month old control (CTRL1), CLN5 heterozygote (CTRL2) and CLN6 homozygote sheep were immunoblotted with an antibody for synaptophysin and GAPDH as loading control. (C) Twenty micrograms of homogenate from occipital and parietal lobe were immunoblotted with an antibody for syntaxin-6 and β-tubulin as loading control.

The expression of metallothionein, a metal-sequestering protein, was increased in the occipital lobe of CLN6 affected sheep (Fig. 2A). The expression of both the 7 kDa form (calculated molecular weight form) and polymerized high molecular forms migrating at approximately 23 kDa and 50 kDa were increased in the affected brain. These higher molecular weight forms have been consistently reported in diverse tissues and animals [31]–[33].

### Synaptic protein changes in CLN6 affected brain

As it is known that synaptic pathology occurs in mice modeling CLN6 disease [34], we hypothesized that synaptic alterations would also be evident in the sheep model of disease. The expression of two important markers of synaptic integrity, synaptophysin and syntaxin-6, were measured. Both were reduced in the occipital lobes of the affected sheep brain when compared to either normal or CLN5 heterozygous controls (Fig. 2B, 2C). Moreover, synaptophysin was also reduced in the parietal lobes of affected animals (Fig 2B). No consistent changes in expression were found in the other brain regions.

### Altered phosphorylation of cellular signaling proteins in the CLN6 affected brain

**Figure 3:**
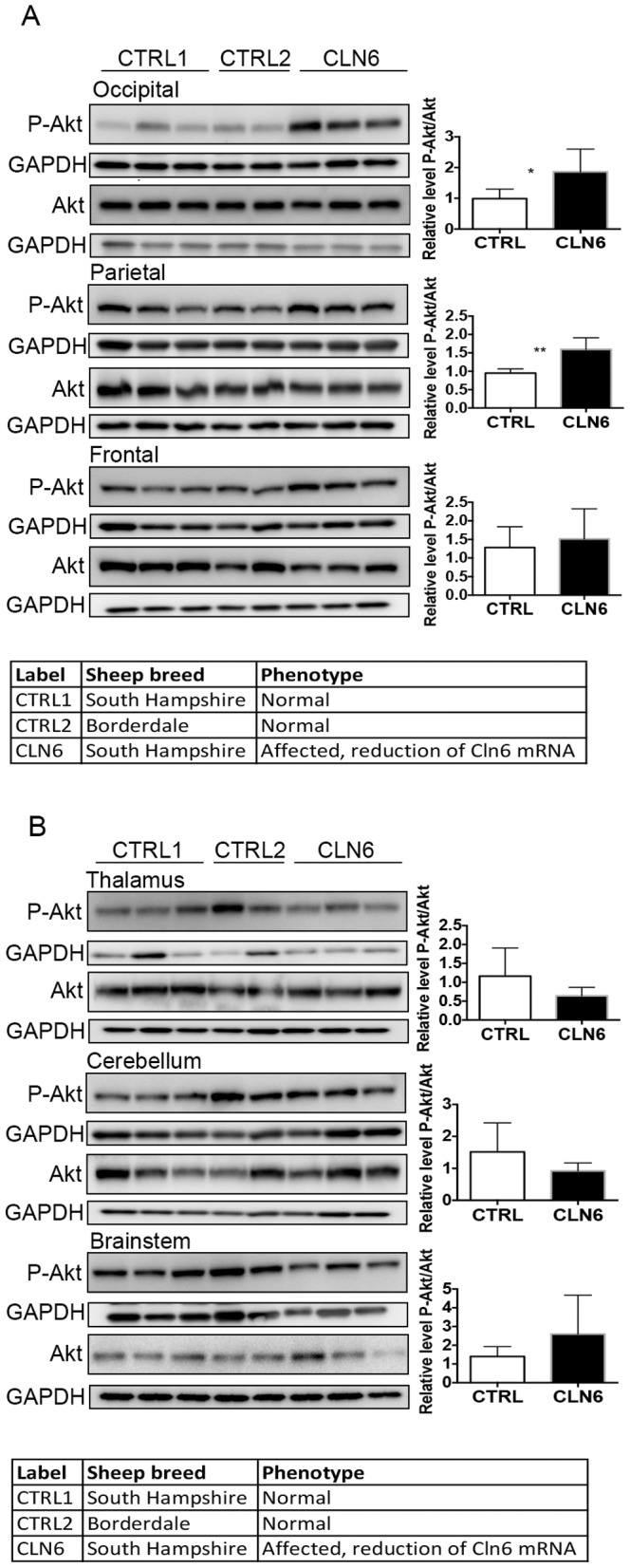
Expression of protein kinase Akt in disease-affected regions of CLN6 sheep brain. (A) Thirty micrograms of homogenate from occipital lobe, parietal lobe and frontal lobe from 12–14 month old control (CTRL1), CLN5 heterozygote (CTRL2) and CLN6 homozygote sheep were immunoblotted with an antibody against phosphorylated Akt and GAPDH as loading control. Fifteen micrograms of homogenate were immunoblotted with an antibody for total Akt and GAPDH as loading control. Densitometric quantification of phosphorylated Akt levels to total Akt was performed as outlined in Materials and Methods. (B) Homogenate from thalamus, cerebellum, and brainstem were immunoblotted with an antibody for phosphorylated Akt, total Akt and GAPDH as loading control as in Figure 3A.

**Figure 4:**
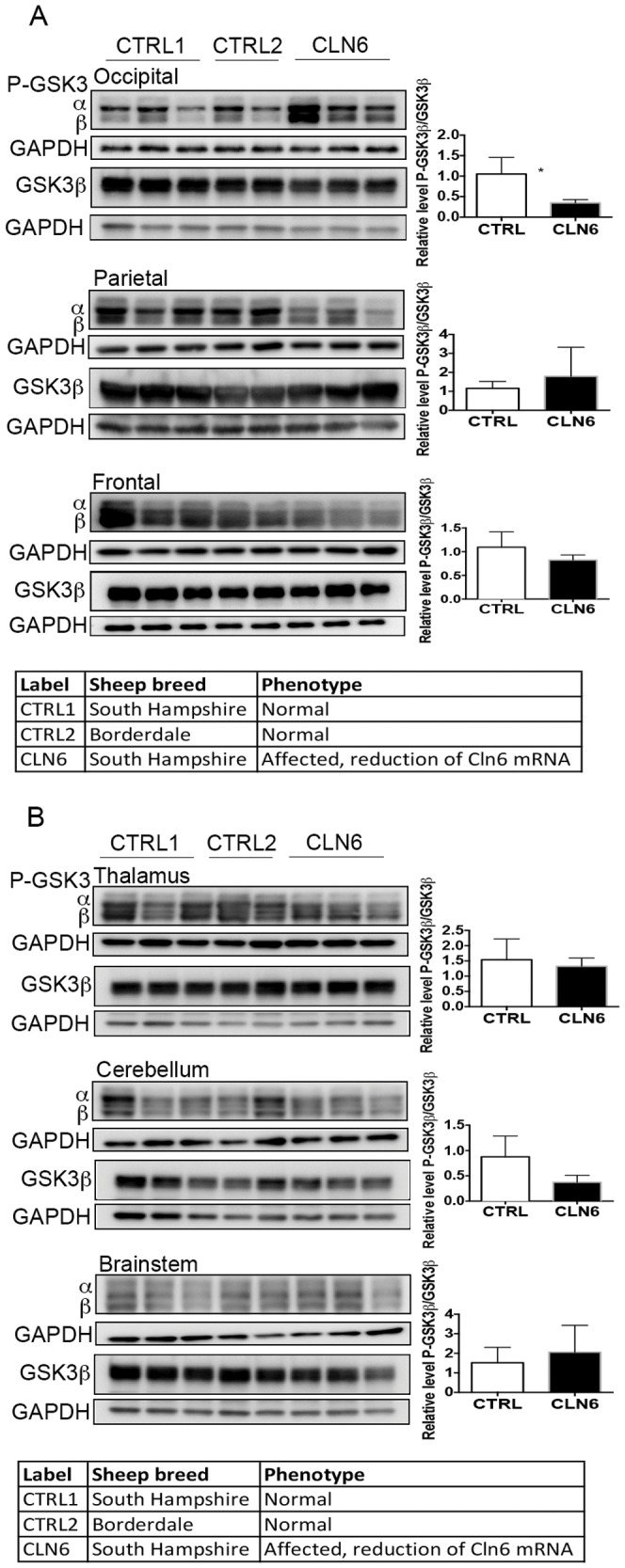
Altered activation of GSK3 in disease-affected brain regions of CLN6 affected sheep. (A) Thirty micrograms of homogenate from occipital lobe, parietal lobe and frontal lobe from 12–14 month old control (CTRL1), CLN5 heterozygote (CTRL2) and CLN6 homozygote sheep were immunoblotted with an antibody against phosphorylated GSK3, and GAPDH as loading control. Twenty micrograms of homogenate were immunoblotted with an antibody for total Akt and GAPDH as loading control. Densitometric quantification of phosphorylated GSK3ß levels to total GSK3ß was performed as outlined in Materials and Methods. Only the phospho-GSKß band was quantified. (B) Homogenate from thalamus, cerebellum, and brainstem were immunoblotted with an antibody for phosphorylated GSK, total GSK3ß, and GAPDH as loading control as in Figure 4A.

The Akt/GSK3 signaling cascade and the MAPK/ERK pathway can be activated by small increases in cytosolic zinc [35], [36]. These pathways were examined by Western blotting to determine any possible relationship to changed zinc concentrations. In particular the activation status of Akt, a protein kinase activated by PI3K that subsequently phosphorylates and inactivates GSK3, was assessed to determine if the Akt/GSK3 signaling pathway was affected by loss of CLN6. An increase in Akt phosphorylation (Ser473) was evident in the occipital and parietal lobes of CLN6 affected sheep (Fig. 3A), but not in the other brain regions (Fig. 3B). To determine if activation of one of the main downstream targets of Akt, GSK3, was also altered in the affected brain samples, total GSK3 and GSK3 phosphorylated at Ser21/Ser9 were assessed. Phosphorylated GSK3ß was reduced in the occipital lobe of CLN6 affected sheep (Fig 4A). Changes were not observed in the other brain regions (Fig.4A, 4B). Taken together, these data suggest that the activation of the Akt/GSK3 signaling pathway is altered in CLN6 affected brains in a region-specific manner.

**Figure 5:**
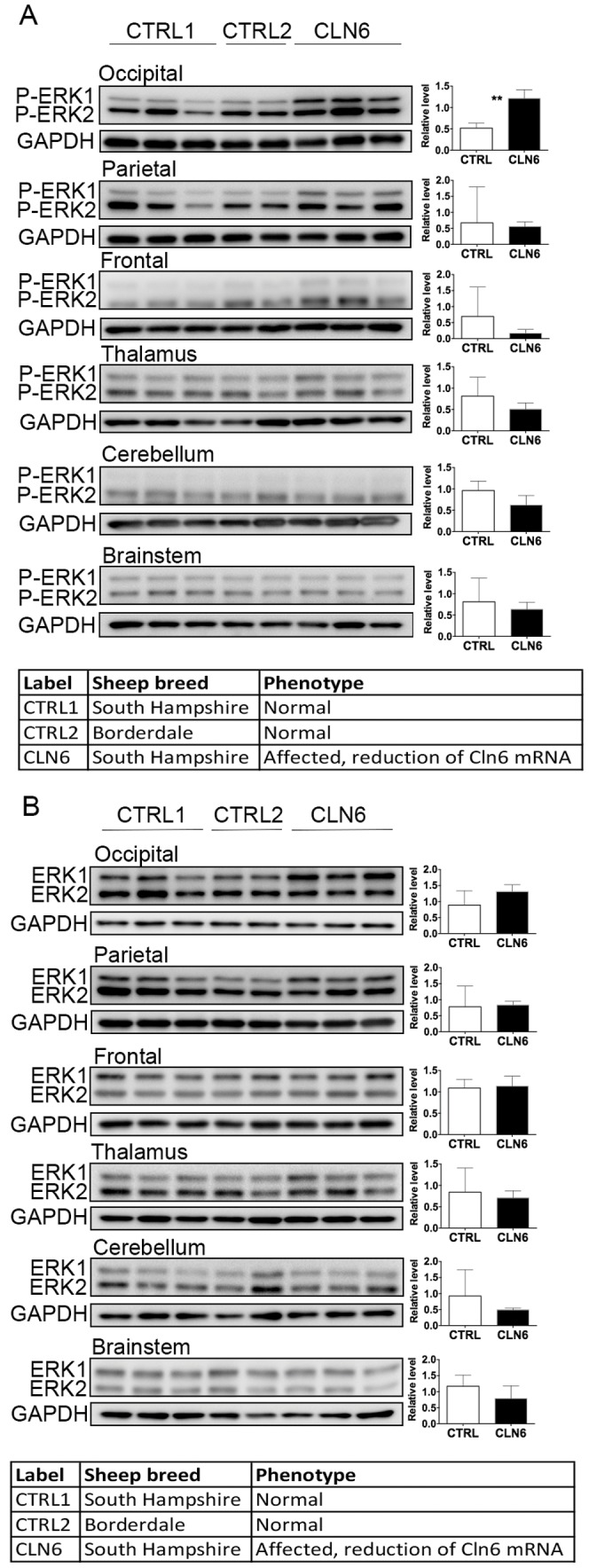
Activation of ERK in disease-affected regions of CLN6 sheep brain. (A) Thirty micrograms of homogenate from occipital lobe, parietal lobe, frontal lobe, thalamus, cerebellum, and brainstem from 12–14 month old control (CTRL1), CLN5 heterozygote (CTRL2) and CLN6 homozygote (CLN6) sheep were immunoblotted with an antibody against phosphorylated ERK1/2 and GAPDH as loading control. (B) Twenty micrograms of homogenates from occipital lobe, parietal lobe, frontal lobe, thalamus, cerebellum, and brainstem were immunoblotted with an antibody for total ERK1/2, and GAPDH as loading control.

Phosphorylation to activate ERK1/2 (phosphorylated at Thr202/Tyr204) increased in the occipital lobes of the affected sheep (Fig. 5A). Total ERK1/2 was unchanged in all of the samples (Fig. 5B) and there was no change to the expression of other members of the MAPK/ERK pathway, such as p38 MAPK or CREB (Fig. S2). In addition, there was no change to oxidative stress related proteins such as heme oxygenase -1 and nuclear factor erythroid 2 related factor-2, or to c-Jun N-terminal kinase, in the CLN6 affected brain (data not shown).

## Discussion

Metal analysis in the ovine CLN6 model of NCL revealed changes to specific metal concentrations in the affected brain. Accumulation of zinc and manganese was evident in those brain regions known to undergo early neurodegeneration [15]. The current study also demonstrated aberrant modulation of cellular signaling pathways, the metal sequestering protein MT, and alterations to synaptic proteins.

The CLN6 affected sheep appear to be normal for the first few months of life until the most notable disease symptom, blindness, becomes obvious at the age of 10–14 months [12], [37]. The mammalian occipital lobe receives and interprets visual sensory messages and contains the primary visual cortex and the parietal lobe is also involved with visuo-spatial processing. These were the two regions that are affected early in ovine CLN6 disease and showed the greatest degree of change in metal concentrations in this study.

While zinc concentrations are not elevated in the cerebrospinal fluid of CLN6 affected sheep (Palmer DN, unpublished data), further studies are required to assess if metal concentrations in the blood of CLN6 affected sheep correlate with those found in the brain. It would also be interesting to determine if brain metal concentrations are elevated at earlier ages. While the data presented here show a significant increase in localized metal concentrations in CLN6 ovine brain, it is not known if this is a driving factor in disease pathogenesis or a consequence of neuropathological changes.

Previous reports suggest concentrations of zinc in the sheep brain to be approximately in the range of 4–10 µg/g, depending on the region studied, and the manganese concentrations in the range of 0.3–0.6 µg/g [38], [39] consistent with the control data here. While there is little knowledge of metal amounts in the brains of sheep affected by disease, it has been shown that zinc and manganese concentrations are elevated by approximately 22% in scrapie-infected sheep [38]. In sheep infected with parasitic psoroptic mange the brain concentration of zinc is reduced by 39% [40]. In the current study, the zinc and manganese concentrations in the occipital lobe of CLN6 affected sheep were increased. Whether these increases are sufficient or prolonged enough to have a subsequent detrimental effect on brain function or are reflective of changes in signaling activity is yet to be established. It is also yet unclear whether the observed changes directly cause neuroinflammation and cell death or whether they occur as a result of these changes.

It should be noted that sub-cellular or localized metal concentrations also vary substantially. Synaptic zinc concentrations are reported to reach 300 µM [41] while major intracellular storage sites for zinc, such as lysosomes may contain zinc at concentrations of ∼300 µg/g tissue or higher [42], [43]. The increases in metal concentrations reported here could reflect substantially larger changes within localized regions or subcellular organelles in the brain. A previous report described the presence of metals including zinc, manganese, copper and iron in lysosome-derived storage bodies isolated from several different ovine CLN6 tissues including the brain [27]. However, the broad range of metal concentrations between tissues did not support a contributory role in storage body formation. Therefore, the sub-cellular localization of increased metal concentrations determined in the present study is unlikely to be associated with storage bodies but may reflect higher concentrations in active subcellular compartments.

Zinc is involved in a variety of neuronal functions, has an important role in synaptic transmission (reviewed in [44]) and serves as a structural component of approximately 10% of cellular proteins [45]. The physiological functions of zinc in the CNS depend on a balance between intra- and extra-cellular zinc concentrations. Because zinc can become toxic at unregulated concentrations these are tightly controlled by a variety of zinc-regulating systems, in which zinc transporters and zinc-sequestering proteins play an important role. Both zinc and manganese share some similar cellular transport systems, including the divalent metal transporter 1 and some of the ZIP transporter proteins [46]–[49]. ATP13A2 is a predicted transmembrane cationic metal transporter, which normally localizes to the lysosome and endosome [50]. When mutated, ATP13A2 mislocalizes to the endoplasmic reticulum [51], [52] where the transmembrane CLN6 protein is also located. Mutations in ATP13A2 have been identified in a familiar form of Parkinson's disease [53] and more recently in NCLs [49], [54], [55] suggesting that metal transporters are important, yet relatively little explored in these diseases.

Metals are sequestered by proteins such as the metallothioneins which regulate intracellular free metal concentrations. The substantial increase in metallothionein in the affected sheep brain coincides with increased metal concentrations in this study. Because zinc-mediated up-regulation of metallothioneins [56] is suggested to be a protective measure against cellular damage it is possible that the increase in metal concentration and metallothionein are linked and serve as an attempt at cellular repair in the affected sheep brain. The fact that the levels of ceruloplasmin, another metal carrier protein implicated in iron homeostasis, correlate with neurological dysfunction in patients with CLN3 disease [57] suggests that metal-binding proteins may play an important role in NCLs.

Metals can activate several cellular signaling pathways. The ERK1/2 and Akt-GSK3ß pathways are important signaling cascades that regulate cell proliferation, migration and survival. Both are also implicated in neuronal death associated with disorders of the CNS [58]. In this study, we have demonstrated that the ERK1/2 and Akt-GSK3ß signaling pathways are altered in the brains of CLN6 affected sheep at the time of observable blindness. These results are consistent with previous observations that alterations to GSK3 activation are a common, important feature of several neurodegenerative diseases [59]. At this point it is not clear whether the observed changes are the cause or consequence of the disease process. While it is interesting to speculate that the local increases in zinc levels in the CLN6 affected sheep may be responsible for the activation of the kinase pathways, further temporal studies are warranted to assess if metal accumulation leads to alterations of cellular phosphatases and sustained ERK1/2 activation in the CLN6 affected brain. Further studies are also required to tease out the precise role of these kinases in the disease process.

Manganese is an essential element that is required for maintaining proper cellular function, yet chronic overexposure of humans to manganese causes manganism, a neurological disease resembling Parkinson's disease [60]. Interestingly, manganism and CLN6 disease share some common pathological characteristics such as cognitive impairment, motor dysfunction and visual impairment [48]. While further studies are clearly required to delineate the consequences of manganese accumulation, it is tempting to speculate that the increase in manganese in the brains of CLN6 affected sheep may be linked to some clinical features of the disease. As manganese accumulation is known to be neurotoxic, it is possible that the increase in manganese concentration in the CLN6 affected brains could be detrimental to the normal function of neurons. Previously it has been reported that levels of manganese superoxide dismutase (MnSOD) are increased in the CLN6 cortex [61]. It remains to be determined if there is an association between the changes in manganese levels in affected brain regions and MnSOD expression or activity.

In addition to zinc and manganese, the concentrations of cobalt, sodium, magnesium and potassium differed in the CLN6 affected brains when compared to controls. Sodium and potassium ions are important for maintaining the neuronal resting and action potentials, and the sodium-potassium pump is the major pump for the exchange of these ions in neurons. The increase in sodium and concomitant reduction in potassium in the CLN6 affected sheep suggests an imbalance in the concentrations of these two metals in the affected brain. It has been reported that the CLN3 protein, associated with a juvenile form of NCL, may interact with the sodium potassium pump on the plasma membrane [62]. The significance of this is unknown but could have implications for control of ion movement in NCLs.

Further to our investigation of metal concentrations, we assessed the expression of synaptic proteins in the CLN6 affected brains, as synaptic loss is a characteristic feature of several neurodegenerative diseases. Synaptic proteins are reduced in the CLN6 form of NCL in those brain regions affected by the pathology. These synaptic changes are likely to reflect the underlying neuronal deterioration associated with neurodegeneration. However, there may be a more direct association between metal alterations and synaptic changes. Our previous studies revealed that altered metal concentrations in the brain are associated with changes to synaptic function and protein expression [63]. Alterations in synaptic proteins also occur early in the pathogenesis of the mouse model of CLN6 disease, the *Cln6^nclf^* mice [34]. SNAP25 and synaptophysin immunoreactivity are reduced in the thalamus of the *Cln6^nclf^* mice at an early symptomatic age and continue to decrease as the disease progresses. Moreover, it has been shown that altered neurite maturation resulting from the loss of CLN6 interaction with collapsing response mediator protein -2 could result in the inability of CLN6 deficient neurons to form new synapses [64]. Our findings of reduced synaptic proteins in the ovine CLN6 model provide further evidence for synaptic vulnerability in NCLs.

Our results provide the first evidence that metal concentrations and the metal-binding protein MT are altered in specific brain regions of CLN6 affected animals and warrant investigation of metal homeostasis in other forms of NCLs. In addition, synaptic changes and activation of cellular signaling pathways were identified in the same brain regions as altered metal concentrations. Ultimately, therapeutic approaches targeted at metals and inhibition of kinases may be feasible for the NCLs.

## Supporting Information

Figure S1 **Metal concentrations in the occipital lobe, parietal lobe, frontal lobe, thalamus, cerebellum, and brainstem of 12–14 month old CLN6 and control sheep were measured using ICP-MS.** The metal concentrations in each brain region are expressed relative to control metal concentrations. Error bars represent S.D.
(EPS)

Figure S2 **Occipital lobe homogenates from 12–14 month old control (CTRL1), CLN5 heterozygote (CTRL2) and CLN6 homozygote (CLN6) sheep were immunoblotted with an antibody for phosphorylated CREB and phosphorylated p38 MAPK.** β-tubulin antibody was used as a loading control.
(TIF)
