# Supplementary figures and images for: Increased Zinc and Manganese in Parallel with Neurodegeneration, Synaptic Protein Changes and Activation of Akt/GSK3 Signaling in Ovine CLN6 Neuronal Ceroid Lipofuscinosis
Source: PLoS One. 2013 Mar 14;8(3):e58644. doi: 10.1371/journal.pone.0058644 (PMC3597713; doi:10.1371/journal.pone.0058644)

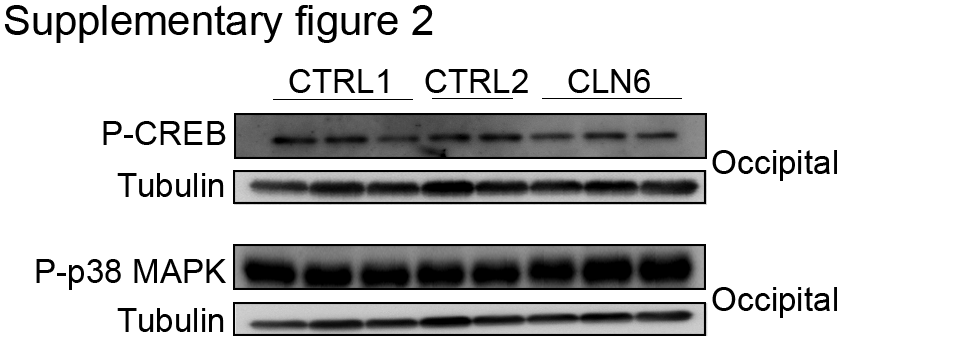

Supplement: Figure S2 — Occipital lobe homogenates from 12–14 month old control (CTRL1), CLN5 heterozygote (CTRL2) and CLN6 homozygote (CLN6) sheep were immunoblotted with an antibody for phosphorylated CREB and phosphorylated p38 MAPK. β-tubulin antibody was used as a loading control. (TIF) [file pone.0058644.s002.tif]
